# Extended-spectrum beta-lactamase and Class 1 integrons in multidrug-resistant *Escherichia coli* isolated from turkeys

**DOI:** 10.14202/vetworld.2019.1167-1174

**Published:** 2019-07-30

**Authors:** Samah Eid, Abdel Hafeez Samir

**Affiliations:** 1 Department of Bacteriology, Reference Laboratory for Veterinary Quality Control on Poultry Production, Animal Health Research Institute, P.O. Box 264, Dokki, Giza 12618, Egypt; 2 Department of Biotechnology, Reference Laboratory for Veterinary Quality Control on Poultry Production, Animal Health Research Institute, P.O. Box 264, Dokki, Giza 12618, Egypt

**Keywords:** Class 1 integrons, *Escherichia coli*, extended-spectrum beta-lactamase, multidrug resistance, turkeys

## Abstract

**Aim:**

This study aimed to investigate the prevalence and implication of extended-spectrum beta-lactamase (ESBL) producing and Class 1 integrons (*int*1) gene carriers *Escherichia coli* isolates that demonstrated multidrug resistance (MDR) phenotypes and was isolated from turkeys that suffered from respiratory manifestations.

**Materials and Methods:**

A total of 120 freshly dead turkey poults that suffered from respiratory manifestations, with a history of treatment failure at Hefna, Belbis, Sharqia (Egypt) were sampled. From each bird lung and liver were aseptically collected and transported for laboratory investigations.

**Results:**

Examination of samples collected from 120 freshly dead turkey poults revealed the isolation of coagulase-positive staphylococci, coagulase-negative staphylococci, *Campylobacter* spp., *Salmonella* spp., *Proteus* spp., *Pseudomonas* spp., *Klebsiella* spp., and *E*. *coli* with the prevalence rates of 12/120 (10%), 30/120 (35%), 17/120 (14.2%), 5/120 (4.1%), 17/120 (14.2%), 6/120 (5%), 7/120 (5.8%), and 18/120 (15%), respectively. *E. coli* isolates were subjected for serotyping and characterization, while the rest of isolates were preserved to be investigated later in further studies. Serogrouping of *E*. *coli* isolates revealed the identification of O119, O6, O8, and O169, while 1/18 of isolates was untypable. Studying phenotypic antibiotic susceptibility profiles of isolates revealed that 18/18 (100%) of isolates demonstrated resistance against cefuroxime, tetracycline, and trimethoprim, 16/18 (88.9%) of isolates demonstrated resistance to amoxicillin/clavulanic acid, enrofloxacin, and norfloxacin, 14/18 (77.8%) of isolates demonstrated resistance to doxycycline and ciprofloxacin, and 9/18 (50%) of isolates showed resistance to gentamycin. Double disk synergy test showed that 6/18 (33.3%), 8/18 (44.4%), and 13/18 (72.2%) of isolates demonstrated the phenotypic pattern of ESBL producers with cefepime, cefotaxime, and ceftriaxone, respectively. Genotypic attributes for beta-lactamase TEM gene and *int*1 gene were studied by reverse transcriptase-polymerase chain reaction and revealed that 17/18 (94.4%) of isolates were positive for both genes. Embryo lethality test indicated that the 18 studied *E. coli* isolates were considered primary pathogens.

**Conclusion:**

The results revealed that 18/18 (100%) of *E*. *coli* isolates demonstrated MDR against three or more antibiotic groups, 9/18 (50%) of isolates showed extensive resistance against the nine tested chemotherapeutic agents from seven antibiotic groups. It is recommended to monitor the circulation of MDR and ESBL-producing pathogens in poultry production in a one health approach, as a preventive measure to mitigate the risk imposed on public health.

## Introduction

*Escherichia coli* is considered one of the normal intestinal microbiota in human and animals including poultry species. In this regard, food safety and quality monitoring measures account on the detection of *E*. *coli* as an indicator of fecal contamination and hygiene throughout the food chain. It is also of significance to monitor the circulation of antibiotic-resistant strains and resistance genes from the animal side including poultry to human side, especially as *E. coli* can play the role of primary or secondary pathogen in a way that imposes a major risk for human health and poultry production.

Recently, extended-spectrum beta-lactamase (ESBL)-producing microorganisms have attracted the global concern in veterinary medicine [1]. ESBL-producing bacteria refer to the ability of those producers to hydrolyze a broad spectrum of beta-lactam antimicrobials as clavulanic acid [2]. In this regard, *E*. *coli* among members of *Enterobacteriaceae* family are considered ESBL producers. ESBLs degrade oxyimino-cephalosporin (cefotaxime [CTC] and ceftazidime) and they are defined as β-lactamases that confer resistance to bacteria against the penicillins, the first-generation, second-generation, and third-generation cephalosporins by hydrolyzing these antibiotics. Thus, ESBL is claimed to be responsible for treatment failure against antibiotics such as CTC and ceftazidime, the most prescribed cephalosporins for the treatment of highly dangerous human infections; consequently, resistance against this group of antibiotics is of high significance. ESBLs are defined as β-lactamases that confer resistance to bacteria against the penicillins, the first, second, and third-generation cephalosporins. Organisms with high ampicillin C (AmpC) activity can give positive ESBL screening since they are active on cephalosporins, whereas they fail to confirm the clavulanic acid screening test as ESBLs can be inactivated by clavulanic acid, while the AmpCs cannot [1]. Contamination of poultry products by ESBL during the slaughtering process was associated with misuse of antibiotics in rearing cycles [3]. Other researchers reported the isolation of ESBL from the gastrointestinal tract of livestock, including turkey flocks [4].

Thus, the present study aimed to investigate the circulation of ESBL-producing integrons one carriers and antibiotic-resistant *E. coli* in turkey flocks and to recommend the most suitable antibiotic treatment.

## Materials and Methods

### Ethical approval

No live birds were used or involved in the study. Freshly dead turkey poults were collected from farms in sterile plastic bags and transported in ice boxes to laboratory within 24 h for examination and testing.

### Sampling

A total of 120 freshly dead turkey poults were sampled to investigate the causative agents and to propose effective antibiotic medication. From each bird lung and liver were aseptically collected and transported for laboratory testing. The reported case history was respiratory manifestations, depression, anorexia, nasal discharge, and high morbidity and mortality rates. The turkey farms are located at Hefna, Belbis, Sharqia (Egypt).

### Isolation and identification of *E. coli* isolates

Samples were investigated for *E*. *coli* isolation according to Barnes and Gross [5], *Salmonella* spp. according to ISO/IEC [6], staphylococci species according to ISO/IEC [7], *Proteus* spp., *Klebsiella* spp., and *Pseudomonas* spp. according to Saif [8], and *Campylobacter* spp. according to ISO [9].

### Serotyping of *E. coli* isolates

Serotyping of *E*. *coli* isolates was applied in the Reference Laboratory for Veterinary Quality Control on Poultry Production, according to Ewing [10].

### Antibiogram

Phenotypic antibiotic susceptibility pattern was studied using antibiotic disk diffusion method according to Quinn *et al*. [11], against nine chemotherapeutic agents from seven antibiotic groups of the most frequently used in the field, as follows: Enrofloxacin (ENR) (5 µg), norfloxacin (NOR) (1.0 µg), amoxicillin/clavulanic acid (AMC) (30 µg), tetracycline (TE) (30 µg), doxycycline (DO) (30 µg), gentamicin (CN) (10 µg), cefuroxime (CFX) (30 µg), trimethoprim (T) (10 µg), and ciprofloxacin (CIP) (5 µg). The results were interpreted according to the criteria recommended by CLSI [12].

### Investigating the presence of ESBL-producing *E. coli*

ESBL-producing phenotype in isolates was investigated by double disk synergy against AMC (30 µg) along with third- and fourth-generation cephalosporin as follows: Cefepime (FEP) 30 µg /fourth-generation, Cefotaxime (CTX) 40 µg/third-generation, and ceftriaxone (CRO) 30 µg/third-generation. The AMC disk was placed at the center of the plate and the three disks were placed at a distance of 1.5 cm. Development of inhibition zone toward the clavulanate disk at 37°C after 24 h incubation was indicative of a potential ESBL positive organism [13]. The isolates that mostly demonstrated phenotypic extensive drug resistance together with phenotypic ESBL pattern were assessed for virulence by the embryo lethality assay (ELA).

### DNA extraction

DNA extraction from samples was performed using the QIAamp DNA Mini kit (Qiagen, Germany, GmbH) with modifications from the manufacturer’s recommendations. Briefly, 200 µl of the sample suspension was incubated with 10 µl of proteinase K and 200 µl of lysis buffer at 56°C for 10 min. After incubation, 200 µl of 100% ethanol was added to the lysate. The sample was then washed and centrifuged following the manufacturer’s recommendations. Nucleic acid was eluted with 100 µl of elution buffer provided in the kit.

### Testing the presence of integrons one gene and ESBL *E. coli* by polymerase chain reaction (PCR)

#### Oligonucleotide primer

Primers supplied from Metabion (Germany) are listed in Table-1. Primers were utilized in a 25-µl reaction containing 12.5 µl of Emerald Amp Max PCR Master Mix (Takara, Japan), 1 µl of each primer of 20 pmol concentrations, 4.5 µl of water, and 6 µl of DNA template. The reaction was performed in an Applied Biosystem 2720 thermal cycler, as shown inTable-1 [14,15].

**Table 1:** Primers sequences, target genes, amplicon sizes, and cycling conditions.

Target gene	Primers sequences (5’- 3’)	Amplified segment (bp)	Primary denaturation	Amplification (35cycles)			Final extension	Reference

				Secondary denaturation	Annealing	Extension		
*bla*_TEM_	ATCAGCAATAAACCAGC	516	94°C 5min	94°C 30 s	54°C 40 s	72°C 45 s	72°C 10min	[14]
	CCCCGAAGAACGTTTTC							
*Int*1	CCTCCCGCACGATGATC	280	94°C 5min	94°C 30 s	50°C 30 s	72°C 30 s	72°C 7min	[15]
	TCCACGCATCGTCAGGC							

*bla*_TEM_=*Beta*-lactamase TEM, *Int*1=Class 1 integrons

### Analysis of the PCR products

The products of PCR were separated by electrophoresis on 1.5% agarose gel (Applichem, Germany, GmbH), in 1× TBE buffer at room temperature using gradients of 5 V/cm. For gel analysis, 20 µl of the uniplex PCR products were loaded in each gel slot. Gelpilot 100 bp and 100 bp plus DNA ladders (Qiagen, Germany, GmbH) and GeneRuler 100 bp DNA ladder (Fermentas, Thermo Scientific, USA) were used to determine the fragment sizes. The gel was photographed by a gel documentation system (Alpha Innotech, Biometra), Germany, and the data were analyzed through computer software.

### ELA

The virulence of *E*. *coli* isolates that demonstrated phenotypic attributes for ESBL was assessed by applying the chick embryo inoculation lethality test, as described by Oh *et al*. [16]. Sixty embryonated chick eggs at 12 days old were used. Ten eggs were inoculated with each of the five tested strains; ten eggs were inoculated with sterile phosphate-buffered saline as negative controls to verify the viability of the chick embryos. The inoculated eggs with the controls were incubated horizontally at 37.5°C and embryos were candled once daily for 7 days post-challenge. The results were calculated with reference to the number of embryo deaths and were interpreted according to Wooley *et al*. [17], who described the primary pathogen as that which causes a mortality rate >29%, moderate virulence pathogen that causes a mortality rate between 10% and 29%, and avirulent pathogen that causes a mortality rate <10%.

### Determining minimum inhibitory concentrations (MICs)/minimum inhibitory concentration (MIC) of ESBL producers

MICs of the two isolates that showed the highest chick embryo lethality was determined in duplicates for each isolate, as described by Andrews and Chih-Cheng *et al*. [18,19]. The MIC was determined against DO, as one of the common field prescribed antibiotics and also due to giving the highest sensitivity rate among the disk diffusion assay applied in the present study. Interpretation of the results of MIC was calculated according to a full range of MIC breakpoints in µg/ml for *E*. *coli* and DO (S<4, I=8, R<16) according to CLSI [12]. The inoculum was prepared by direct colony suspension method, and MIC is defined as the lowest concentration of antibiotic that prevented macroscopically visible growth after 18 h of incubation. Minimum bactericidal concentrations (MBCs) were determined by subculturing 100 μL from each well with no visible growth on Trypticase Soy Agar. MBC is defined as the lowest concentration of DO that prevented absolute growth on subculture after 18 h of incubation. MBC is identified by determining the lowest concentration of antibacterial agent that reduces the viability of the initial bacterial inoculum by a pre-determined reduction such as ≥99.9%. The MBC is complementary to the MIC, whereas the MIC test demonstrates the lowest level of antimicrobial agent that greatly inhibits growth, the MBC demonstrates the lowest level of an antimicrobial agent resulting in microbial death [12].

## Results

Examination of total 120 freshly dead turkey poults suffered from respiratory manifestations revealed the isolation of coagulase positive staphylococci, coagulase negative staphylococci, *Campylobacter* spp., *Salmonella* spp., *Proteus* spp., *Pseudomonas* spp., *Klebsiella* spp., and *E*. *coli* with the prevalence rates of 12/120 (10%), 30/120 (35%), 17/120 (14.2%), 5/120 (4.1%), 17/120 (14.2%), 6/120 (5%), 7/120 (5.8%), and 18/120 (15%), respectively. *E*. *coli* isolates were subjected for serotyping and characterization, while the rest of isolates were preserved to be investigated latter in further studies, as shown in Table-2.

**Table 2:** Prevalence rate of isolates.

Isolates	Number of isolates	Prevalence rate isolates (%)
*Pseudomonas* spp.	6/120	5
Coagulase-positive staphylococci	12/120	10
Coagulase-negative staphylococci	30/120	35
*Escherichia* *coli*	18/120	15
*Salmonella* spp.	5/120	4.1
*Proteus* spp.	17/120	14.2
*Klebsiella* spp.	7/120	5.8
*Campylobacter* spp.	17/120	14.2

Serogrouping of *E*. *coli* isolates with regard to the somatic O antigen revealed that O119 was the most prevalent serogroup 6/18 with a rate of 33.3%, followed by O6 and O8, each with a detection rate of 4/18 (22.2%), O169 3/18 (16.7%), while 1/18 (5.6%) isolate was untypable, as shown in Table-3.

**Table 3:** Serotyping of *Escherichia* *coli* isolates.

Identified serotypes	No. of isolates	Serotype distribution rate (%)
O6	4/18	22.2
O119	6/18	33.3
O8	4/18	22.2
O169	3/18	16.7
Untypable	1/18	5.6
Total	18/18	100

Phenotypic antibiotic susceptibility profile of isolates was studied by disk diffusion, the result revealed that 18/18 (100%) of isolates demonstrated resistance against CFX, TE, and T, 16/18 (88.9%) of isolates demonstrated resistance against AMC, ENR, and NOR, 14/18 (77.8%) of isolates demonstrated resistance to DO and CIP, and 9/18 (50%) of isolates showed resistance to CN, the results also showed that 18/18 (100%) of isolates demonstrated multidrug resistance (MDR) against chemotherapeutic agents belonged to more than 3 antibiotic groups, 9/18 (50%) of isolates showed extensive resistance against the nine tested chemotherapeutic agents that belonged to seven antibiotic groups, as shown in Table-4.

**Table 4:** Phenotypic susceptibility patterns of *Escherichia* *coli* isolates.

Antibiotic group	Chemotherapeutic agent	Resistance rate	Sensitivity rate
Penicillins	Amoxicillin/clavulanic acid	16/18 (88.9%)	2/18 (11.1%)
Cephalosporins	Cefuroxime	18/18 (100%)	0%
Aminoglycosides	Gentamicin	9/18 (50%)	9/18 (50%)
Tetracycline	Tetracycline	18/18 (100%)	0%
	Doxycycline	14/18 (77.8%)	4/18 (22.2%)
Quinolones	Enrofloxacin	16/18 (88.9%)	2/18 (11.1%)
	Norfloxacin	16/18 (88.9%)	2/18 (11.1%)
Diaminopyrimidine	Trimethoprim	18/18 (100%)	0%
Fluoroquinolone	Ciprofloxacin	14/18 (77.8%)	4/18 (22.2%)
(Second-generation quinolone)			

*In vitro* susceptibility (double disk synergy test) of ESBL to beta-lactam antibiotics, the results showed that 6/18 (33.3%), 8/18 (44.4%), and 13/18 (72.2%) of isolates demonstrated phenotypic pattern of ESBL producers by showing inhibition zones toward the central disk of AMC with FEB, with CTC, and with CRO, respectively, as shown in Table-5.

**Table 5:** Results of studying *Escherichia* *coli* isolates for ESBL phenotypic activities.

Antibiotic agent	Number of sensitive isolates	Detection rate (%)
Cefepime	6/18	33.3
Cefotaxime	8/18	44.4
Ceftriaxone	13/18	72.2

ESBL=Extended-spectrum beta-lactamase

**Figure-1:**
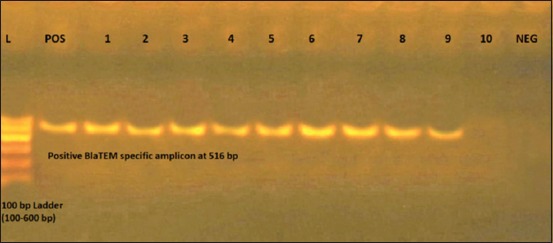
From the left to the right: Lane L: 100 bp ladder (100-600 bp), POS: Positive *bla*_TEM_ control at 516 bp, Lanes (1-9): Specific amplicon at 516 bp, Lane 10: Negative result no amplicon, NEG: Negative control.

**Figure-2:**
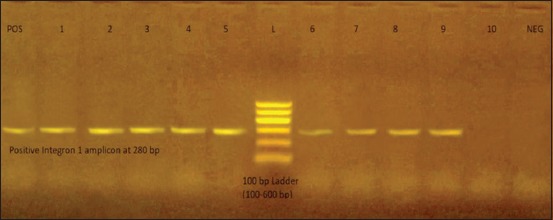
From the left to the right: POS: Positive integron 1 control at 280 bp, Lanes from (1-9): Specific amplicon at 280 bp, Lane L: 100 bp ladder (100-600 bp), Lane (10): Negative result no amplicon.

Testing the presence of beta-lactamase TEM (*bla*_TEM_) gene and Class 1 integrons (*int*1) gene in *E*. *coli* isolates by PCR revealed that 17/18 (94.4%) of isolates were positive for the studied genes, as shown in Table-6 and Figures-1 and 2.

**Table 6:** Results of PCR detection of target genes.

Target gene	Detection rate
*bla*_TEM_	17/18 (94.4%)
*Int*1	17/18 (94.4%)

PCR=Reverse transcriptase polymerase chain reaction

*E*. *coli* isolates that demonstrated phenotypic attributes for extensive drug resistance, ESBL producing, and confirmed genotypic carriage for *bla*_TEM_ and *int*1 genes were selected for virulence assessment by ELA; the results revealed that isolate number 5 (O119) and isolate number 6 (O119) showed 100% lethality; the results also revealed that all isolates involved in the study were considered primary pathogens, as resulted in at least 60% embryo lethality. The isolates that showed 100% lethality (two isolates) were selected for MIC determination, as shown in Table-7.

**Table 7:** Results of ELA.

Isolate number	Serotype	Number of death	Lethality rate (%)	ELA
3	O6	6/10	60	Primary pathogen
5	O119	10/10	100	Primary pathogen
6	O119	10/10	100	Primary pathogen
7	O8	8/10	80	Primary pathogen
8	O169	8/10	60	Primary pathogen

ELA=Embryo lethality assay

MIC of DO (1-32 μg/ml) was studied against the two tested isolates (number 5 and number 6), the result demonstrated that isolate number 5 was considered susceptible, as the detected MIC breakpoint was <4 µg/ml, while the isolate number 6 was considered intermediate resistant as the detected MIC breakpoint was = 8 µg/ml, Table-8.

**Table 8:** Determining MIC of *Escherichia* *coli* isolates.

MIC	1	2	4	8	16	32	Interpretation
Isolate no 5	+	+	−	−	−	−	Sensitive
Isolate no 6	+	+	+	−	−	−	Intermediate resistance

MIC=Minimum inhibitory concentration, +=Turbidity, −=No visible turbidity

Antibacterial agents are usually regarded as bactericidal if the MBC is no more than 4 times the MIC. The detected MBC for isolate number 5 was 8 µg/ml, while the MBC for isolate number 6 was 16 µg/ml, Table-9.

**Table 9:** Determining MBC of *Escherichia coli* isolates.

MBC	1	2	4	8	16	32
Isolate no 5	5×10^3^	3×10^2^	2×10^2^	0	0	0
Isolate no 6	4×10^4^	4×10^3^	5×10^2^	2×10	0	0

MBC=Minimum bactericidal concentration

## Discussion

In the present study, the bacteriological examination was applied to investigate the causative agents that are most probably implicated with the field problem reported in turkey flocks in Hefna, in winter 2018. Other studies attributed severe course of diseases with respiratory manifestations and high mortality rates in turkeys to concurrent infections with *Klebsiella pneumoniae* subsp. *pneumoniae*, *E*. *coli*, and *Pseudomonas* *fluorescens;* other researchers also recorded the isolation of *Campylobacter jejuni* from the liquid cecal content of diseased birds [20]. More studies identified that avian pathogenic *E*. *coli*, *Staphylococcus aureus*, and *Ornithobacterium rhinotracheale* were the three most frequently diagnosed pathogens of turkeys [21]. In the same instance, *Salmonella* spp. were isolated from turkeys with a prevalence rate of 31/98 (10.3%) [22]. Furthermore, scientific studies reported that poultry is implicated as a source of transmission of zoonotic MDR *Proteus*, which is considered the second causative agent of human urinary tract infection, consequently could be a problem for both veterinary and public health sectors [23]. In the same regard, *Pseudomonas* was isolated from diseased broilers suffered from respiratory manifestations, septicemia, and high mortality with a prevalence rate of 30% [24]. In the current study, *E*. *coli* isolates were characterized, while other bacterial isolates were preserved to be investigated in further studies.

Although *E*. *coli* is considered a member of the intestinal microbiota in vertebrates, including poultry, *E*. *coli* is one of the leading causes of economic losses in turkey production [25]. Moreover, *E*. *coli* are characterized by the presence of virulence genes encoding for important virulence factors as fimbriae, adhesion, toxins, siderophores, capsule, hemolysins, and invasion. Commensal, nonpathogenic *E*. *coli* may have a maximum of three virulence genes while avian enteropathogenic *E*. *coli* usually have tens of virulence genes [26]. Colibacillosis could be caused by a primary *E*. *coli* infection or secondary *E*. *coli* infection predisposed by viral and mycoplasma infection, or environmental stress factors, in which the birds suffered from respiratory manifestation, systemic infection, and probably fatal septicemia. The prevalence rate of *E*. *coli* recorded in the current study was 18/120 (15%) of the total examined birds, while higher prevalence rate of avian pathogenic *E*. *coli* isolated from necropsied turkey poults was recorded by other studies, 13/15 (86.7%) [27]. Other researchers also recorded higher prevalence rate 6/6 (100%) of *E*. *coli* from turkey products [28].

It is of significance to identify the circulating serotypes implicated with recorded field cases in a particular geographic area and a chronologic period, thus may contribute to the epidemiological study, control and treatment recommendations. Studies applied on turkey colibacillosis cases reported the isolation of O18 and O111 [29], the identification of O78 and O111, and untypable isolates were also reported in other studies [30]. In the present study, serogrouping of *E*. *coli* isolates with regard to the somatic O antigen revealed that O119 was the most prevalent serogroup 6/18 (33.3%), followed by O6 and O8 each with a detection rate of 4/18 (22.2%) and O 169 3/18 (16.7%), while 1/18 (5.6%) isolate was untypable.

The indiscriminate use of antibiotics in food animals including turkey flocks may select for resistant bacteria capable of causing human and animal diseases. Moreover, the circulation of resistant bacterial pathogens in farmed food animals including poultry may increase the risk of transmission of bacterial infections to human through food, direct or indirect contact between birds and human.

Phenotypic antibiotic susceptibility patterns of *E*. *coli* isolates were studied by disk diffusion and revealed that 18/18 (100%) of isolates demonstrated resistance against CFX, TE, and T, 16/18 (88.9%) of isolates demonstrated resistance against AMC, ENR, and NOR, 14/18 (77.8%) of isolates demonstrated resistance to DO and CIP, and 9/18 (50%) of isolates showed resistance to CN. Similarly, other studies detected resistance against CTC, ampicillin, and TE with rates of 31/33 (94%), 33/33 (100%), and 28/33 (85%), respectively [28]. Resistance phenotypes to CTC was recorded by other studies, with a rate of 30/80 (37.5%), with the conclusion that the majority of *E*. *coli* isolates were expressing ESBL MDR to amoxicillin, AMC, ceftazidime, and CTC with demonstration of positive synergy in 23/80 (29%). Moreover, the isolates showed MDR, not only to beta-lactams but also to CIP, TE, and T/sulfamethoxazole [31]. It was also reported that 95.8% of APEC isolates showed resistance to TE, and DO, and penicillin and demonstrated resistance rates of 47.9%, 6.2%, and 6.2% to T, ENR, and CIP, respectively, but demonstrated full susceptibility to cephalosporin [32].

The current study also showed that 18/18 (100%) of isolates demonstrated MDR against at least one chemotherapeutic agents belonged to three and more antibiotic classes. The study also revealed that 9/18 (50%) of isolates showed extensive resistance against all the tested chemotherapeutic agents that belonged to seven antibiotic groups. In this regard, scientific studies reported the detection of ESBL *E*. *coli* isolates that demonstrated MDR against three and more antibiotic classes at least for one agent with multidrug patterns mostly demonstrated against (aminoglycosides, penicillin, TE, fluoroquinolones, and sulfonamides) in 96.1% and 82% of APEC isolates [32,33].

In human medicine, two compartments have been suggested as habitats of ESBL-producing bacteria, one is nosocomial infections in hospitals and one is in the community as a result of consumption of contaminated food [34].

Furthermore, high frequency of ESBL-producing *E*. *coli* was isolated from broiler carcasses in Germany, Netherlands, and Spain [1,35]. In the present work, studying ESBL phenotypic attributes by double disk synergy technique revealed that 6/18 (33.3%), 8/18 (44.4%), and 13/18 (72.2%) of isolates demonstrated positive phenotypic pattern of ESBL producers by showing inhibition zones toward the central disk of amoxicillin/clavulanic acid and FEB, CTC, and CRO, respectively. Studies with similar purposes were applied in turkey products and detected ESBL-producing *E*. *coli* with a prevalence rate of 6/6 (100%), with a recorded resistance rate of 30% to ceftazidime [28]. Similarly, ESBL-producing *E*. *coli* were isolated with rates of 11/45 (24%) and 6/20 (30%) from breeder farms [36].

Genotypic coding for ESBL producers include the genotypic attributes for *bla*_CTX-M_, *bla*_TEM_, and *bla*_SHV_ genes, in the present study investigation for the presence of *bla*_TEM_ gene was studied by PCR, the results demonstrated that 17/18 (94.4%) of isolates were positive for *bla*_TEM_ gene. Other studies targeted the detection of *bla* genes (*bla*_CTX-M_, *bla*_TEM_, and *bla*_SHV_) in *E*. *coli* isolates from poultry origin revealed the detection of *bla*_TEM_ gene solely, and in concurrence with other *bla* genes members [31,36]. Moreover, high detection rates of *bla*_TEM_ gene (70%, 100%) were reported [33,36].

Antibiotic resistance has been greatly linked to the dissemination of linked genes encoding resistance inserted in mobile genetic elements, mainly integrons. In the present study, integrons Class 1 gene was detected by PCR in 17/18 (94.4%) of isolates, other study reported the detection of integrons of Class 1 in two isolates while detecting integrons of Class 2 in ten isolates, the same study also reported the disagreements of their recorded low detection rate of integrons Class 1 gene versus integrons Class 2 due to the results of many researches that concluded the dominance of integrons of Class 1 in animal-derived *E*. *coli* or in animal products as well as human isolates [37]. Lower detection rates of integrons Class 1 gene in *E*. *coli* isolates from turkey broilers and chicken breeders were recorded by other researchers (0.9% and 12.5%) [32,36].

In the current study assessment of *E*. *coli*, isolates were of concern as a tool to associate their isolation to the reported field cases in the turkey farms in Hefna, Belbes, Sharkia (Egypt). Moreover, the virulence assessment was also required to assist the selection among the MDR isolates for use in determining MIC and MBC to recommend the most suitable antibiotic treatment.

In this regard, the study applied an ELA, as considered similar to intravenous, subcutaneous, and intratracheal challenge models and is capable of discriminating between virulent and avirulent avian *E*. *coli* isolates. Furthermore, ELA is regarded as sensitive and specific for virulence assessment in diagnostic laboratories [16,38]. The current observations revealed that 5/5 (100%) of the tested isolates were considered as primary pathogens as O6, O8, and O169 as they contributed to 60% embryo lethality rate, while the two tested O119 caused 100% embryo lethality rate.

Based on ELA results, the MIC and MBC for DO were applied to the two tested O119 isolates which resulted in the highest observed ELA (100%) and the results indicated that DO has shown therapeutic effectiveness as one of the tested isolates demonstrated susceptibility and showed no viability with MIC at 4 and MBC at 8, while the other isolate demonstrated intermediate resistance with MIC 8 and MBC 16. *In vitro* study of DO and DO combinations effectiveness on MDR *E*. *coli* has the conclusion that although DO is an ancient and cheap antimicrobial agent, it exhibits a broad-spectrum effect against pathogens including Gram-negative and considered useful and even drug of choice in the treatment of MDR *E*. *coli* and other MDR pathogens [19].

## Conclusion

Although penicillin and cephalosporins are considered the drugs of choice in poultry industry, the use of third and fourth generations cephalosporins, aminoglycosides and fluoroquinolones in veterinary medicine are believed to give rise for antimicrobial-re­sistant bacterial strains that are implicated in economic losses in poultry production. Furthermore, impose the risk of treatment failure. Consequently, it is important to investigate whether ESBL producing E. coli and class 1 integrons carriers E.coli of poultry ori­gin represent a zoonotic risk. Thus, monitoring and surveillance of antimicrobial resistance circulation in one health approach are recommended. Moreover, raising awareness for the appropriate use of antibiotics in both veterinary and human medicine is of importance.

## Authors’ Contributions

SE designed the study, collected the samples, and applied bacteriological examinations, wrote the manuscript. AHS applied the PCR testing. SE and AHS approved the final manuscript.
